# The old friends hypothesis: evolution, immunoregulation and essential microbial inputs

**DOI:** 10.3389/falgy.2023.1220481

**Published:** 2023-09-12

**Authors:** Graham A. W. Rook

**Affiliations:** Centre for Clinical Microbiology, Department of Infection, UCL (University College London), London, United Kingdom

**Keywords:** immunoregulation, evolution, microbiota, inflammation, socioeconomic status, natural environment, epithelial barrier, biodiversity

## Abstract

In wealthy urbanised societies there have been striking increases in chronic inflammatory disorders such as allergies, autoimmunity and inflammatory bowel diseases. There has also been an increase in the prevalence of individuals with systemically raised levels of inflammatory biomarkers correlating with increased risk of metabolic, cardiovascular and psychiatric problems. These changing disease patterns indicate a broad failure of the mechanisms that should stop the immune system from attacking harmless allergens, components of self or gut contents, and that should terminate inappropriate inflammation. The Old Friends Hypothesis postulates that this broad failure of immunoregulation is due to inadequate exposures to the microorganisms that drive development of the immune system, and drive the expansion of components such as regulatory T cells (Treg) that mediate immunoregulatory mechanisms. An evolutionary approach helps us to identify the organisms on which we are in a state of evolved dependence for this function (Old Friends). The bottom line is that most of the organisms that drive the regulatory arm of the immune system come from our mothers and family and from the natural environment (including animals) and many of these organisms are symbiotic components of a healthy microbiota. Lifestyle changes that are interrupting our exposure to these organisms can now be identified, and many are closely associated with low socioeconomic status (SES) in wealthy countries. These insights will facilitate the development of education, diets and urban planning that can correct the immunoregulatory deficit, while simultaneously reducing other contributory factors such as epithelial damage.

## 1. Introduction

It was noted as early as 1873 that the prevalence of hay fever was increasing amongst wealthy urban populations but not amongst farmers (1). Increases in allergic disorders accelerated during the 20th century (2, 3), and this focused attention on Th2 responses. However it was soon realised that the prevalences of autoimmune disorders and inflammatory bowel diseases (IBD) that involve different effector pathways of the immune system were often rising in parallel at the same time and in the same places (2, 4, 5). This suggested a broad failure of immunoregulation allowing multiple branches of the immune system to target harmless allergens, autoantigens and gut contents. Moreover even in individuals with no obvious inflamed target organ, systemically raised levels of inflammatory biomarkers were increasingly being observed in wealthy developed countries (6), and correlated with an increased risk of metabolic, cardiovascular (7) and psychiatric disorders (8–10).

These observations point to a broad failure of regulation of the immune system. Therefore the recent increases in chronic inflammatory disorders are likely to be attributable to recent changes in the stimuli that drive the development of immunoregulatory mechanisms. Expansion of the repertoire of effector lymphocytes of the immune system is driven by microbial inputs, but so is expansion of the regulatory anti-inflammatory arm that censors inappropriate immune responses and terminates redundant inflammation (11). This is true in the gut which has received most attention (12, 13), but it is equally true for the skin (14, 15) and the lungs (11). The mechanisms involved are outlined in a later section.

**Figure 1:**
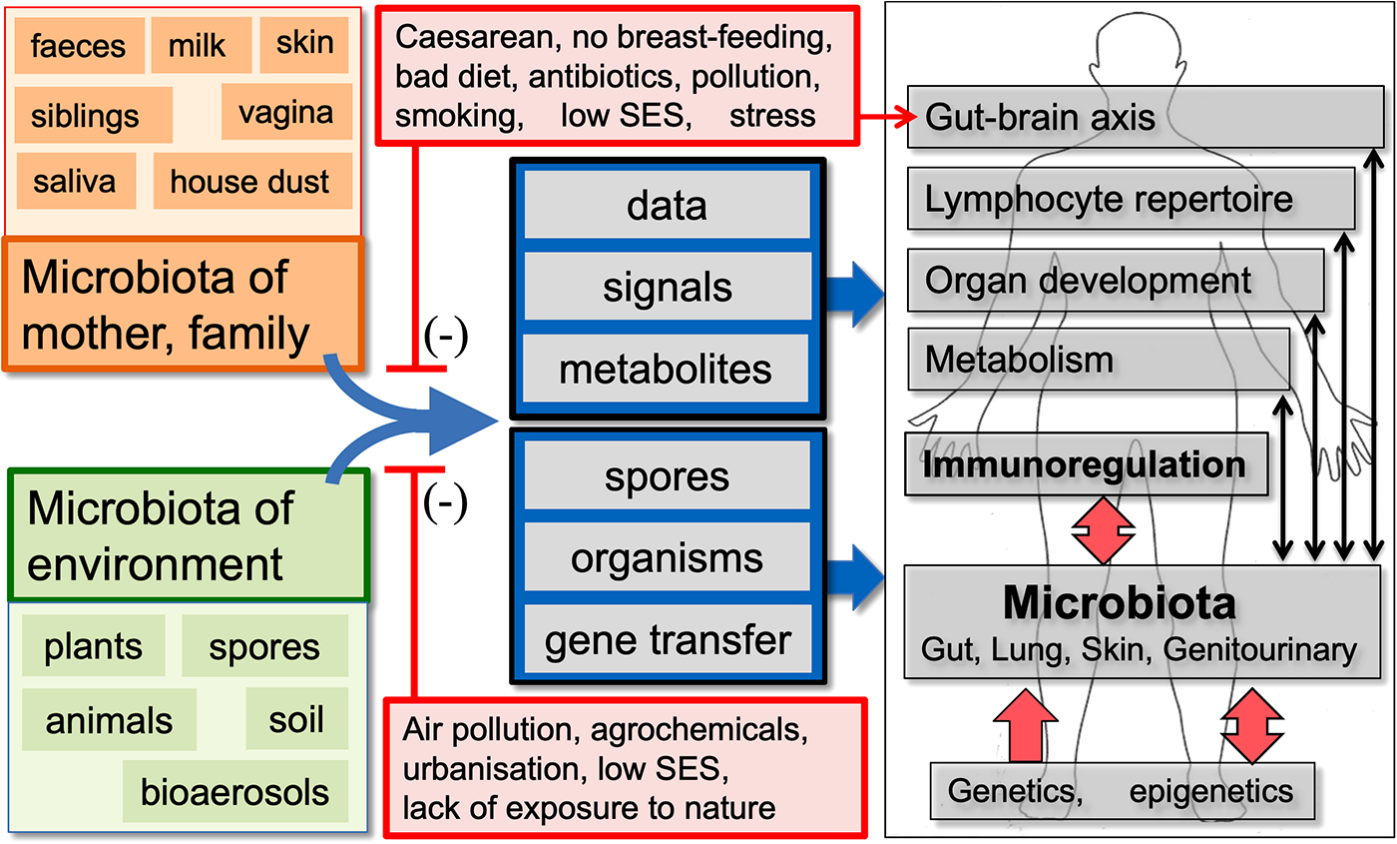
A summary of the old friends hypothesis. We co-evolved with the microbiota of mother, family and the natural environment, which are involved in multiple physiological functions and provide essential data and signals that drive development of the effector and regulatory arms of the immune system. Factors that distort these microbiota and/or decrease our exposure to the microbiota-derived signals and metabolites that drive immunoregulation may be contributing to the increase in chronic inflammatory disorders. In some human communities many, but not all, of the factors that distort microbial inputs are associated with low socioeconomic status (SES). (Infections and vaccines that enhance “Trained Immunity” rather than immunoregulation are not shown, but are discussed in the text.)

The “Old Friends Hypothesis” therefore uses an evolutionary framework to identify the microbial inputs that drive immunoregulatory circuits (16, 17). These are likely to be found amongst organisms with which humans co-evolved as hunter-gatherer omnivores and on which we may be in a state of evolved dependence (18). This paper outlines the evolution of our relationship to microorganisms, and the evolution of the immune system, and then considers many broad categories of microorganism, with particular attention to their role in driving immunoregulation and in allergic disorders. But no hypothesis in biology is ever the whole answer. The Old Friends Hypothesis should be considered together with the need for biodiversity in the microbial input (19), and the increased risks of allergic disorders when epithelia are exposed to substances driving “danger signals” and increased permeability (20–22) and Figure 1.

## 2. Evolution from and with microorganisms

Cellular life forms came into existence about 3.8 billion years ago. Approximately 1.5 billion years later an endosymbiotic event led to an organism similar to an alpha-proteobacterium living inside another organism, where it evolved to become the mitochondrion (23). This appears to have happened only once and led to the evolution of all eukaryotic life forms. About 65% of human genes have their origins in Bacteria, Archaea and eukaryotic microbes (24), including genes responsible for synthesizing neurotransmitters in the brain (25).

So we evolved from microorganisms but we also evolved in a world dominated by them. Calculating the biomass of major life kingdoms in terms of carbon content reveals that bacteria rank second after plants in total biomass, comprising approximately 7 gigatons of carbon compared to humans who only constitute 0.06 gigatons. Additionally, there are around 10^30^ bacteria, archaea, and fungi present on earth, outnumbering humans by a ratio of 10^20^ to 1. The symbiotic microbiota in our guts is at least as abundant as our own human cells, and they produce more than ∼30% of the small molecules present in our peripheral blood, affecting our physiology in largely unexplored ways (26). These gut organisms were initially separated from host tissues by a chitin barrier, which persisted in arthropods and annelids. In chordate invertebrates such as tunicates, the barrier persists and is embedded in a mucin gel. In the most primitive vertebrates, a more substantial mucus layer is produced, and in mammals, the chitin layer is absent permitting the complex mucus layers to interact with microorganisms. Some organisms adhere to the mucus, are nourished by it, and regulate the function of underlying cells (27). This is reminiscent of the situation in plants, where molecules secreted by the roots attract and nourish microorganisms that engage in symbiotic nutrient exchange and signaling (28).

The gut microbiota has co-evolved with humans. Comparing human and ape microbiota to calculate the date when humans and the great apes diverged from a common ancestor yields a date similar to that indicated by comparisons of their chromosomal or mitochondrial DNA (29). Moreover many bacterial and at least one archaeal strain have co-diversified and spread across the planet with their human hosts. These organisms are becoming “obligate symbionts”, losing many genes they would need to survive outside the human gut (30). They are co-evolved components of our physiology.

### 2.1. Co-evolution of the immune system and microorganisms

How did the immune system cope with this massive co-evolving community of diverse microbial species in close contact with the host tissues? The innate immune system uses inherited pattern recognition receptors (PRR) to recognise conserved microbial components. However, pathogens can evolve quickly and produce structures that are not recognized by existing PRRs. The innate immune system can try to adapt by duplicating and modifying PRR genes, but this process is slow and results in a cluttered genome. The adaptive immune system in vertebrates solves this problem by using somatic hypermutation to create a large variety of receptors with little increase in genetic complexity. This involves mutating the genes that encode B and T lymphocyte receptors to produce a diverse array of clones. While this reduces the genetic load, it also creates new problems such as the production of useless or autoreactive lymphocytes. However, since each clone expresses only one receptor, any cell line that recognises nothing or that recognises the host's own tissues can be eliminated. Most autoreactive cells are eliminated in the thymus which expresses self-antigens. However in order to select and retain lymphocyte clones that recognise a diversity of microorganisms, and that can manage and tolerate a diverse microbiota while eliminating pathogens, the adaptive immune system must obtain data from microbial inputs, acquired mostly from mother, family and the environment (31). Thus the immune repertoire of each new individual is matched to the microbial environment into which that individual was born.

## 3. What do microbial exposures do for the immune system

It is evident from the previous paragraph that the developing immune system requires data from microbial inputs. However this is only one of many essential functions of microbial inputs, many of which have profound effects on the regulatory arm of the immune system. These are listed in Table 1 and in the following sections.

**Table 1:** Some microbial inputs and their functions.

Input	Effect on immune system	Refs.
Data		
Diverse microbial epitopes	Select diverse repertoire of useful lymphocyte clones. Expand lymphocyte repertoire to recognise novel pathogens	(31–33)
Microbial epitopes from microbiota transported to thymus	Select lymphocyte clones that recognise gut microbiota Tolerate symbiotic partner organisms	(34)
Biodiversity, organisms		
Diverse organisms	Populate microbiota. Drive organ development. Set up regulation of metabolic and immune systems	(35–37)
Spores	Trapped by mucus and cilia, swallowed. Expand or restore the gut microbiota. Treg-inducing strains.	(38)
Low dose pathogens	Disarm in airways, swallow. Immunity to common pathogens. “Trained Immunity”	(39, 40)
Metabolites		
Short chain fatty acids from fermentation of fibre, polysaccharides	Inhibition of histone deacetylases (HDACs) and activation of G-protein-coupled receptors (GPCRs). Anti-inflammatory effects. Increased Treg activity	(41, 42)
Bacterial tryptophan catabolites	Indoles and indolepropionic acid promote differentiation of Treg via the aryl hydrocarbon receptor (AhR)	(43)
Secondary bile acids	Less Th17 cells and enhanced production of RORγ + Treg Anti-inflammatory effects. Increased Treg activity.	(12, 44)
Branched chain amino acids	Maintain Treg. Enhance immunoregulation	(45)
Signals		
Microbial components (e.g. some LPS & muramic acid derivatives)	Signals via Pattern Recognition Receptors (PRR) Tolerance, immunoregulation, innate immune system regulation. Drive release of *TNFAIP3* (A20)	(46–49)
DNA	Horizontal gene transfer. Adapt strains to gut ecosystem and adapt metabolic repertoire to diet	(50–52)
Extracellular vesicles and microRNA		
Microbial extracellular vesicles and miRNA	Influence promotion by dendritic cells of T cell differentiation into various effector types or Treg	(53)
Pathogens and vaccines		
Infection; crowd infections of childhood or other pathogens	Death or immunity to the pathogen. “Trained Immunity” after some infections or during persistent infection. Epigenetic modulation of adaptive immune system	(54–57)
Vaccines	Immunity to the pathogen. Some live vaccines also provide non-specific survival benefit via “Trained Immunity”	(58–60)
Other inputs		
Bacteriophages from people or environment	Regulate composition of gut microbiota. Release of inflammatory components by lysis. Depletion of anti-inflammatory strains when phage imbalance?	(61–63)

### 3.1. Data

An input of microbial antigens and epitopes is needed in early life to select the mutated lymphocyte clones that need to be retained as memory cells. All life forms are constructed at least in part from variants of the same building blocks that evolved long ago in early microorganisms (24, 25). This is important because it means that if individual humans or animals are exposed to a sufficiently diverse range of microbial epitopes, the lymphocyte clones that are retained and expanded will, by chance, contain some that recognise viruses or other pathogens to which the individual was never previously exposed such as HIV or COVID-19 (31–33). This point emphasises the need for exposure to microbial biodiversity (19). Interestingly, biodiversity of the gut microbiota has a strong correlation with health (64). Even declining health in old age is associated with a decrease in gut microbiota biodiversity (35, 65). However other possible functions of biodiversity are suggested in the next paragraph.

### 3.2. Biodiversity

It may not be only the diversity itself that is important but also the increased likelihood of having essential species that drive functions such as immunoregulation (discussed later) (36,37), or other undiscovered necessary functions. For example, a single protein synthesised by a single organism is required for the expansion of the pancreatic β cells in the zebra fish (66). We do not know if there are similar hidden requirements in the human microbiota because we cannot recolonise germ-free humans with one species at a time in order to identify such dependencies.

Another possibility is that complex ecosystems are more stable. Species diversity can protect ecosystems from excessive damage caused by environmental change because having many species increases the chance that some can quickly adapt to the new conditions (67).

Biodiversity also prevents dangerous biofilm formation. An organism's physiology changes when it forms biofilm. Some organisms become more pathogenic, resistant to the immune system and even resistant to antimicrobials. Much of the pathology caused by

*Candida albicans* occurs when it switches from yeast to hyphal forms during biofilm formation (68). In patients with IBD, gut microbiota can penetrate the mucus barrier and form biofilm that adheres to the epithelial surface. Bacteria from the microbiota of healthy donors do not cross human intestinal epithelial cell monolayers *in vitro* but organisms from the biofilm can do so (69). High biodiversity of the gut microbiota may affect quorum sensing signals and prevent the switch to biofilm.

### 3.3. Metabolites that modulate immunoregulation

The roles of the major gut microbiota-derived metabolites in the regulation of immune function were reviewed recently (12) and will be outlined very briefly here and in Table 1. Short chain fatty acids (SCFA) such as acetate, propionate and butyrate, derived from the microbial fermentation of dietary fibre, can enhance production of IL-10, TGF-β and Treg (12, 41). Secondary bile acids generated by the microbiota, notably derivatives of lithocholic acid, can downregulate Th17 cells and enhance production of RORγ + Treg (12, 44). Several bacterial tryptophan catabolites including various indoles and indolepropionic acid promote Treg differentiation via the aryl hydrocarbon receptor (AhR) (12, 43). Branched-chain amino-acids such as valine, leucine and isoleucine are present in the diet, but are also generated by the microbiota, and maintain Treg (45).

The Treg populations in the skin are attracted and expanded by signals from skin microbiota (15). Lipases from components of this microbiota (*Corynebacterium, Staphylococcus,* and *Micrococcus*) act on lipids secreted by sebaceous glands to generate butyrate and other SCFA (70), which have immunoregulatory properties as outlined above. Moreover, some authors suggest that ammonia-oxidising bacteria (AOB) and archaea that colonised human skin in the past converted the high levels of ammonia and nitrate in human sweat into nitrite and nitric oxide which entered through the skin and exerted immunoregulatory functions (71), notably, downregulation of Th2 responses (72). Because these organisms are exquisitely sensitive to alkylbenzene sulfonate detergents this may no longer occur, though clinical trials applying AOB to the skin are being performed (73).

### 3.4. Microbial components and signals that modulate immunoregulation

Exposures to microbial components that activate PRRs such as TLR4, TLR2, TLR9 or AhR or PI3K/Akt/mTORC1 signaling systems drive the establishment of immunoregulatory mechanisms. These microbial components transiently trigger inflammation, but repeated low dose exposures may prime anti-inflammatory mechanisms due to the release of IL-1β which can induce tolerance to itself and to endotoxin (LPS) (74). Therefore exposure to these microbial signals informs the immune system about the nature of the microbial environment and triggers immunoregulatory epigenetic adjustments. For example, LPS induced Treg via tolerogenic dendritic cells and TGF-β in an animal model (75). LPS in dust in the farming environment may protect against allergic responses by inducing A20 in lung epithelial cells (76). A20 is a potent inhibitor of the NF-κB signaling pathway and its expression is increased in Amish farmers using traditional farming methods (46, 47).

In mice, administering a TLR2 agonist resulted in a decrease in Th17 cells and an increase in type 1 regulatory T cells in the spleen. This led to a reduction in the severity of Experimental Autoimmune Encephalomyelitis (EAE) (77). The same researchers discovered that patients with Multiple Sclerosis (MS) had significantly lower levels of a TLR2 agonist derived from bacteria in their blood compared to healthy individuals (77).

TLR9 is an intracellular PRR that recognizes CpG motifs that are not methylated. These motifs, common in microbes, typically trigger an inflammatory response. However, some variants of CpG motifs and other microbial DNA sequences may have anti-inflammatory properties (78, 79). This appears to be the case for many species of *Lactobacillus* (80) which could explain why the probiotic effects of lactobacilli depend on the presence of TLR9 in the gut (81).

DNA is exchanged by horizontal gene transfer (HGT) both between different gut-resident microorganisms, and between gut microbiota and organisms in the environment (50–52). This exchange can occur between species that diverged in an evolutionary sense in the distant past, and constitutes a global network of gene exchange that can help strains to adapt rapidly to new diets and metabolic needs (51, 52). It is not clear whether HGT modulates the organisms that drive development of immunoregulatory mechanisms.

### 3.5. Extracellular vesicles and microRNA: a 2-way dialogue

Host-derived microRNAs (miRNA) in membrane-bound extracellular vesicles (EV) can modify microbial gene expression. Mice that lack the miRNA-processing enzyme, Dicer, cannot form miRNAs. Such mice developed abnormal gut microbiota and exacerbated colitis, but administrating faecal miRNA corrected these abnormalities (82). So the host regulates gene expression in the gut microbiota. But this type of communication is 2-way. Recent work has shown that microbiota-derived EV have profound physiological effects on host metabolism, on the regulation of the immune system and on Treg numbers (53).

### 3.6. Bioaerosols

The microbial diversity of air is comparable to that of seawater, soil, and the human gut (83). Moreover, the lungs and airways constitute a sense organ with cellular sensors that can detect biogenic aerosols in inhaled air whether derived from microorganisms or from other sources such as plants. Plant polyphenols like quercetin, resveratrol and curcumin can reduce inflammation through the AhR (84). Microbial pigments such as phenazines and naphthoquinones can also regulate inflammation and anti-bacterial responses (48). All of these molecules and others from algae and higher plants can inhibit protein kinases of the PI3K/Akt/mTORC1 signaling system, which is believed to have an anti-inflammatory effect (49). This is likely to be relevant to the immunoregulatory benefits of exposure to the natural environment discussed later.

## 4. Which are the microbial exposures that matter

With this evolutionary, developmental and pharmacological background we can begin to ask which microbial exposures are the ones that drive the immunoregulatory mechanisms that seem to be deficient in modern developed countries.

### 4.1. Crowd infections of childhood

The seminal observation that hay fever was less prevalent in children with older siblings (85) led initially to speculation that modern domestic hygiene was reducing exposure to the common infections of childhood leading to imbalances within the immune system. However this hypothesis was unlikely because humans cannot be in a state of evolved dependence on these infections. They are mostly “crowd infections” that could not have persisted in isolated ancestral hunter-gatherer groups. Measles, for example, probably did not hit human populations until late in the Roman empire when appropriately large populations existed (86–88).

The debate was rapidly resolved when epidemiological studies revealed that these crowd infections of childhood do not protect from allergic disorders (89–91), and often trigger or exacerbate them (89, 92, 93).

We now understand that the protective effect of older siblings, while unquestionably correct, is likely to be due to increased transmission of the microbiota of mother and of the natural environment, as discussed later (94, 95). This does not mean that the common infections of childhood have no effect on the immune system but their effect, apart from inducing immunity to themselves, is non-specific activation of the innate immune system (“Trained Immunity”) rather than amplification of down-regulatory anti-inflammatory pathways, as briefly explained in the next section.

#### 4.1.1. “trained immunity”

In the 1930s, Pullinger observed that infecting cattle or guinea pigs with *M. tuberculosis* conferred resistance to *Brucella abortus* (96). Pullinger and subsequent authors attributed this to nonspecific activation of monocytes (96, 97). Further studies showed cross-protection between unrelated parasite species, and between *Listeria monocytogenes* and influenza virus (98). This work was largely forgotten until it was reported in the 1980s that live vaccines such as measles, polio, smallpox, and the Bacillus of Calmette and Guérin (BCG) could enhance resistance to unrelated infections (58, 59). These effects are mediated by epigenetic modulation of several components of the innate immune system, including natural killer cells and monocytes as Pullinger and Elberg had suggested (60, 99). “Trained Immunity” is not known to enhance the immunoregulatory pathways that are deficient in rich urban communities, but in view of the interconnected nature of all aspects of the immune system it is likely that changing patterns of Trained Immunity will be found to be relevant (100). This constitutes a major gap in our knowledge.

### 4.2. Mother and other people

The transfer of the co-evolved human microbiota (29, 30) from mother (and siblings) to infant is critical for the development of the infant's microbiota, as well as the immune and metabolic systems (94). Certain lifestyle factors, such as Caesarean deliveries, lack of breastfeeding, poor diet (discussed in greater detail below and in Table 2), antibiotic use, and insufficient mother-infant intimacy, can reduce this transfer, and are associated with an increased risk of immunoregulatory disorders (94, 95, 101). Some organisms in the child's microbiota appear later and continue to accumulate until 5 years of age (131). These organisms are probably acquired from other family members and at day-care centres, as well as from the natural environment. Microbial strains can be transmitted person-to-person through normal social and mother-infant interactions both within and outside the home (132, 133). Such transfers may be diminished by modern lifestyles.

**Table 2:** Low socioeconomic status (SES) and distortion of microbial exposures.

Disruptors of required microbial exposures		Effects	References
Modern delivery and neonatal care	Caesarean deliveries	Delayed development of mature microbiota in neonate. Reduced milk-derived prebiotics	(94, 95, 101)
	Lack of breast-feeding		
Pollution	Traffic, air pollution	Direct effects on microbiota, and indirect effects via host immune system and damaged epithelia	(28, 102, 103)
	Agrochemicals		
	Damp, sick-building	Toxic microbial secondary metabolites	(104–107)
	Exposure to cleaning and hygiene products	Epithelial damage. Th2 adjuvant effects	(20–22)
Lack of green space	Little exposure to strains and spores from nature	Low biodiversity of microbiota Less immunoregulatory strains Increased psychiatric disorders Metabolic/cardiovascular disorders	(108–111)
	Less sunlight, vitamin D	Defective immunoregulation, altered microbiota	(112, 113)
Stressors	Drug abuse, violence, heat, noise, sleep disorders	Changes to microbiota and reduced biodiversity via signals within the gut-brain axis	(114–116)
Poor diet	Unvaried	Low biodiversity of microbiota	(64, 117)
	Ultraprocessed: emulsifiers, excitotoxins,	Leaky gut, neuronal damage. Deficient micronutrients	(118, 119)
	Low fibre	Low short chain fatty acids (SCFA)	(12, 41, 120)
	Low vitamins	Potential deficiencies	(121)
	Obesity	Metabolic problems Cardiovascular problems	(42, 122)
	Sugars, artificial sweeteners	Distorted microbiota. Raised glycaemic response	(123)
Education	Smoking	Switch from aerobes to anaerobes, biofilm and *Clostridioides difficile*	(124)
	Antibiotic misuse	Exposure *in utero* or in early life correlates with metabolic and immunoregulatory problems	(125–127)
		Reduced deconjugation of sex hormones, reduced re-uptake, distorted sexual maturation	(128) [Discussed and referenced in (129)]
	Vaccine hesitancy and refusal	Infection risk and lack of beneficial non-specific vaccine effects	(130)

### 4.3. The home

Can exposure to the microbiota of modern homes be considered essential? If we think in terms of evolution the response must be that it depends on the home. In the past, humans lived in natural shelters such as caves, or constructed homes from natural materials like stones, mud, branches, and leaves. Later more sophisticated homes were constructed by rearranging natural materials such as timber, stone, straw, soil, clay, animal dung, thatch or turf. The microbial makeup of such homes would have been similar to that of the surrounding natural environment. Even when damp and decaying, the organisms present would have been those with which humans co-evolved. However, modern homes made from synthetic materials, biocide-treated timber, plywood, and synthetic gypsum board harbour a microbiota that is different from that of the natural environment (134, 135). This difference is more pronounced in urban homes that are distant from nature (136). Furthermore, when a modern home is damp and deteriorating, as is often the case in households of low Socioeconomic Status (SES), the bacterial and fungal microbiota can produce secondary metabolites that are hazardous to human health, leading to varying degrees of “Sick Building Syndrome” (104–137) and increased likelihood of children being hospitalized for respiratory infections (107)*.* Therefore, it is improbable that the unnatural microbiota of modern homes, especially when the home is of low SES, provides necessary or desirable microbial exposure for infants. However, when the microbiota of homes is similar to that of farms and the natural environment, it can be beneficial, particularly for disorders related to faulty immunoregulation like asthma, as discussed later (138–140).

#### 4.3.1. Cleaning agents: are our homes too clean?

**Figure 2:**
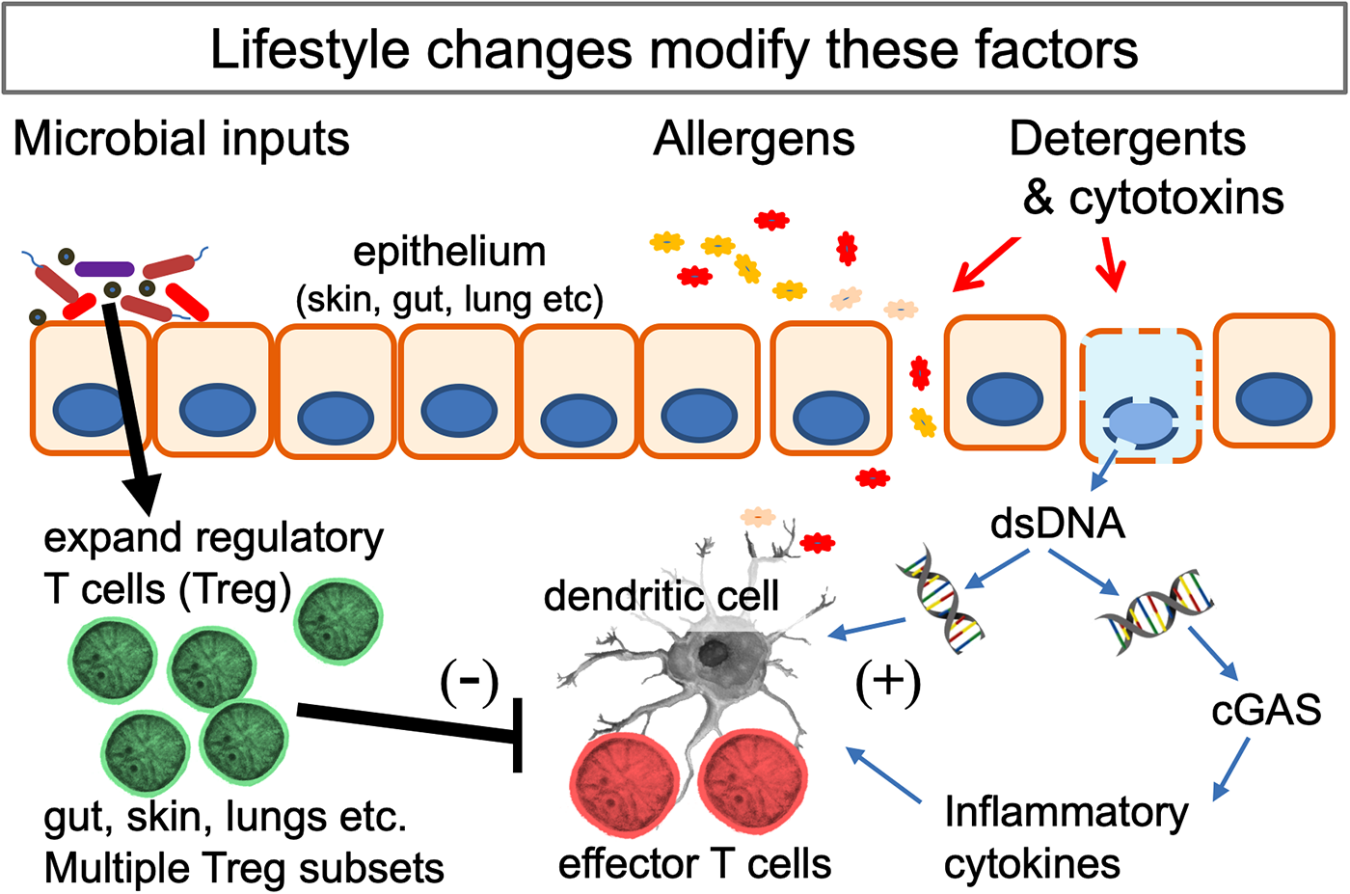
Interactions of the mechanisms behind several hypotheses that seek to explain the increase in allergic disorders. Multiple microbial signals and metabolites (listed in Table 1) expand the population of Treg at epithelial surfaces. Lifestyle changes (listed in Table 2) can reduce and distort these signals. Potential allergens cross the epithelial barrier when this is damaged. The likelihood of a Th2 response is increased if cell death releases double-stranded DNA (dsDNA) which enhances the function of dendritic cells and, when detected by the dsDNA-detector cGAS (cyclic GMP-AMP synthase), drives release of inflammatory cytokines. The lifestyle and environmental changes involved can all be considered as examples of gene-environment mismatch in modern urban societies.

Another relevant variable is the use of cleaning agents. The media have publicised the notion that the increase in allergic disorders might be attributable to reduced microbial exposures caused by excessive personal or domestic cleanliness (141). However epidemiological studies seeking correlations between the use of cleaning agents and allergic disorders have produced wildly discordant results (142, 143). We have suggested elsewhere that this might be due to a failure to take into account a probable effect of exposing infants to aerosols of cleaning agents (22). Cleaning agents, especially when used as sprays, have been thought to have harmful effects on the lungs of adult cleaning personnel who are exposed to them every working day (144). Detergents cause increased epithelial permeability and cytotoxins cause local cell damage that provides “danger signals” and activates allergic defence mechanisms in the airways or gut (20, 21, 145, 146). For instance, food antigens usually cause tolerance, but if the antigen is detected in the gut in the presence of cell death an allergic Th2 response may be generated (20). This antigen then becomes a proxy for the cytotoxic molecule (which is often not itself immunogenic), so subsequent exposures will trigger an allergic reaction, even if the cytotoxin is absent. Exposure to detergents and cytotoxins is most likely in homes of low SES where infants are crawling in confined spaces while their mothers use trigger sprays containing potentially toxic cleaning products. In a UK cohort, where use of cleaning agents correlated with wheeze and atopic eczema (142), it was noted that the most intense use of chemical household products correlated with low educational level, smoking, and poor, crowded housing (147). The infant airway in which immunoregulatory balance is being established, is likely to be very sensitive to these exposures. In animal models successful Th2 adjuvants cause some cytotoxicity and release of double stranded DNA (dsDNA) (146) which enhances antigen presentation by MHC Class II (148). Moreover dsDNA activates the local immune system via the airway dsDNA sensor (cGAS; cyclic GMP-AMP synthase) pathway (149), which is essential for the induction of airway allergy (150). Further details of this pathway can be found here (151) and in Figure 2.

### 4.4. Natural environment

In addition to the microbiota of mother and family, our evolving hunter-gatherer omnivore ancestors were inevitably exposed to the microbiota of the natural environment and of animals that were hunted, eaten or domesticated. During the late 19th century Blackley noted that farmers were less prone to hay fever than were people residing in urban areas (1). Since then, numerous studies have supported the notion that early-life exposure to a farming environment can reduce the prevalence of allergic disorders (46, 138). Some of the protective effect appears to come from early life exposure to farm animals (152) or dogs (139, 153) which cause measurable changes to the gut microbiota (154). Furthermore, living close to green spaces can also decrease the risk of allergic sensitization (155). Some of these studies have revealed immunological evidence that strongly suggests a cause-and-effect relationship, rather than a mere coincidental association (46, 47, 155). For example, Amish farmers who use traditional farming techniques have a very low incidence of allergic disorders and were found to have increased biomarkers of immunoregulation, compared to industrialized Hutterite farmers (46, 47). Similarly, exposing children to natural biodiversity in their school playgrounds in a controlled clinical trial resulted in an increase in peripheral blood biomarkers of immunoregulation (109). The evidence supporting the protective effect of exposure to the microbiota of the natural environment is less complete for other chronic inflammatory disorders, but there is suggestive evidence for IBD (156), autoimmune diseases (38) and psychiatric disorders (110) and metabolic and cardiovascular diseases (111).

#### 4.4.1. Soil

Which organisms from the natural environment are important in addition to those from animals? Although there is no direct evidence linking soil consumption to health benefits, it is evident that soil is a significant source of microbial exposure in natural settings. Soil microorganisms become airborne in dust during dry conditions, but also when raindrops impact the soil, because tiny explosions of soil organisms occur, releasing them into the air (157). Additionally, soil organisms settle on food, particularly in farmers' markets where washing and packaging are minimal. Moreover geophagy (consumption of soil) is an evolved behaviour. It is probable that all vertebrates, especially in early life, engage in it, and the green iguana is a well-studied example of this (158). Many primate species, including gorillas, orangutans, and chimpanzees have been observed eating soil (159, 160). Geophagy is a common practice in many cultures (160, 161), and is frequently observed during pregnancy, not only in underdeveloped rural societies but also in Western cultures where it is often considered a pathological manifestation of pica. Many of the Treg-inducing and immunoregulation-enhancing strains of bacteria are found in soil (13, 162).

##### 4.4.1.1. Bacteriophages

There about 10^9^ phages/gm of soil so vast quantities are taken in every day in food and drinking water. Bacteriophages are the most numerous biological entities in the gut and constitute about 95% of the gut virome. They influence the composition of the microbiota and the turnover of susceptible species, and therefore regulate the release of pharmacologically active microbial components and metabolites (61, 63). The overall effect on immunoregulation is poorly documented but there is a report of low diversity of gut bacteriophages in children who develop the autoantibodies implicated in type 1 diabetes (T1D), or the clinical disease (163). It is possible, therefore, that contact with appropriate bacteriophages from mother, other people and the natural environment influences immunoregulation.

##### 4.4.1.2. Spores

Soil is also a major source of spores which are particularly relevant to immunoregulation. Spores are resilient and can survive in the environment for centuries [reviewed in (164)]. It appears that around 60% of bacterial genera in the gut can produce spores, including some that were not previously known to do so (165, 166). Spores play a vital role in transmitting strictly anaerobic organisms essential to human health from one person to another through the environment, since the spores are not killed by oxygen (165, 166). Therefore spore-forming anaerobic organisms are probably among the components of a child's microbiota that appear later in infancy and continue to accumulate until the age of 5 (131, 166). Many of these organisms are critical because they make SCFA which have numerous essential physiological roles (12, 41), and promote expansion of the Treg population (13, 38) as outlined in Table 1.

### 4.5. Low dose pathogens

Inevitably there are sometimes pathogens in respired air, but very low doses typically lead to protective immune responses rather than disease. When bacteria attach to the nasal mucosa, released exosomes may transfer inducible nitric oxide synthase to neighbouring epithelial cells and increase release of nitric oxide (167). Pathogens also trigger the release of cathelicidin, and other human antimicrobial peptides (AMP) of which there are more than 100 (39). When bacteria and cathelicidin enter the cell the NLRP3 inflammasome is activated and a cascade of events is initiated, including the activation of caspase 1, the death of infected cells, and the release of pro-inflammatory cytokines IL-1β and IL-18. These events enhance inflammation and recruit neutrophils which are induced to form networks of extracellular fibres consisting mainly of DNA called Neutrophil Extracellular Traps (NET). These NETs contribute to inactivation of microorganisms (39).

Ultimately, these mechanisms in the airways kill or disarm the respired organisms, which are then taken up by the lymphoid tissue of Waldeyer's ring or exposed to acid in the stomach before being sampled by the dendritic cells in the small bowel (40). In this way, inspired low doses of pathogens may provide useful data to the immune system and prime immunity to potential pathogens, but they do not appear to be crucial for setting up immunoregulation.

### 4.6. Sequence of exposures

The order in which live or killed vaccines are given can determine the nature of their non-specific effects (59), indicating that the order in which vaccines are given determines the epigenetic changes that they induce. This may also apply to the sequence in which infections are experienced (168). For example it has been suggested that acute lymphocytic leukaemia (ALL) may result from delayed exposure to an agent that, during human evolution, would have been encountered in very early life (169). Interestingly ALL is more common when early life exposure to microorganisms is reduced, such as after Caesarean delivery, lack of breastfeeding and lack of older siblings (169). This is similar to risk factors for immunoregulatory disorders like allergies (94, 95).

A similar concept has been proposed to explain the increase in T1D that seems to have appeared in parallel with the development of the modern Western lifestyle. The prevalence of T1D fell after the introduction of rotavirus vaccines administered at 2 months (170).

### 4.7. Other infections

It was reported 20 years ago that hay fever and asthma were less frequent in subjects seropositive for hepatitis A virus (HAV), *Toxoplasma gondii*, and herpes simplex virus 1 (171). It was not clear whether these infections were markers of plentiful food-borne and orofaecal microbial exposure to family and environment, or whether the infections were themselves enhancing immunoregulation. Some of these are persistent infections which deserve to be considered because they may drive immunoregulation in order to limit immunopathological damage to the host. A few particularly obvious examples of infections that exert non-specific effects on the immune system are considered briefly below.

#### 4.7.1. Helicobacter pylori

*Helicobacter pylori* was carried by humans for much of our evolutionary history. However, the use of antibiotics has reduced the seroprevalence of *H. pylori* to less than 10% among native-born citizens in Western urbanized countries. This could be relevant because epidemiological surveys have shown an inverse relationship between *H. pylori* seropositivity and childhood asthma (56), and experiments in mice indicate that *H. pylori* drives expansion of Treg subsets expressing CXCR3 or RORγt and demethylation at the FOXP3 locus (172).

#### 4.7.2. Herpes viruses

More than 90% of adults have been infected with at least one of the five most common species of Herpes virus (HSV-1, HSV-2, varicella zoster, Epstein–Barr virus, cytomegalovirus). These viruses tend to remain latent but periodic reactivation can influence the state of the immune system. Mice that were latently infected with either murine gammaherpesvirus 68 or murine cytomegalovirus were found to be resistant to bacterial pathogens such as *Listeria monocytogenes* and *Yersinia pestis* (55). This resistance is attributed to intermittent reactivation followed by cytokine-mediated activation of macrophages. We are unaware of evidence that these viruses protect significantly from allergic disorders.

#### 4.7.3. Tuberculosis

Latent tuberculosis infection (LTBI) is very common in developing countries and the persistent presence of these organisms has been demonstrated in multiple tissues (173). There is a small risk of developing clinical tuberculosis. However, treating LTBI in non-HIV-infected individuals does not provide a survival benefit because it increases mortality from other causes (57). Similarly BCG, a live vaccine derived from a mycobacterium, appears to protect the elderly from respiratory virus infections (174). In other words LTBI and BCG vaccine both induce “Trained Immunity” outlined earlier. But do they induce immunoregulation? Some early studies suggested an inverse correlation between tuberculin test positivity and atopic disorders (175) but this did not prove cause and effect. Many studies have subsequently investigated whether BCG vaccination protects from allergies (176). The conclusion is that there can be a weak protective effect if the BCG is given to neonates, particularly in babies at greater risk because of atopic disorders in the parents (177), but the effects are small.

#### 4.7.4. Helminths

The rise in inflammatory disorders has been attributed to the decline in helminth infections because helminths drive immunoregulatory mechanisms to protect the host from fatal immunopathology (178). Some authors propose that we have evolved to rely on this background immunoregulation so that the absence of helminths results in an excessively inflammatory immune response (179). It was logical to postulate evolved dependence on helminths and this author has endorsed it in the past but it now seems more likely that adaptation of the developing immune system to the presence of helminths was largely epigenetic and is lost after a few generations without helminths. Various helminth species reside in distinct sites, such as blood, tissues, bladder, or gut, and each species damps down inflammation through a unique mechanism (178). Additionally, helminth burdens can vary widely between individuals, even when they live in comparable geographical areas. As a result, there is no “inevitable” helminth-related factor that would have driven the evolution of a permanent germ-line-encoded adaptation leading to evolved dependence [discussed in (180)]. Intermittent environmental factors or infectious stresses are managed through reversible epigenetic adaptations, not via germline encoded mutations which would lead to frequent gene-environment mismatch. These epigenetic mechanisms explain occasional reports of lower prevalence of allergic symptoms in children who were infected with geohelminths in early life [discussed in (178)] but meta-analyses suggest that overall, helminthiases are associated with increased allergic manifestations probably driven by the powerful Th2 responses that they evoke (181).

This probable role of epigenetics also helps us to understand the conflicting results of helminth therapy trials in MS. It is reported that when Argentinian MS patients become naturally infected with helminths they would have encountered during childhood, disease progression can be halted (182). In early life their immune systems developed in the presence of these helminths and consequent epigenetic adjustments necessitate their continued presence. However, in regions where helminths have not been endemic for multiple generations, trials of helminth therapy for MS or other autoimmune disorders have been disappointing (183–185). While evolution turns the inevitable into a necessity, it allows the intermittent or temporary to become an option through epigenetic adjustments. In the absence of helminths in Europe and the USA the need for them has faded.

##### 4.7.4.1. Anecdotal evidence of efficacy may be valid

There is a wealth of anecdotal evidence for the efficacy of self-administered therapy with a variety of helminths. However helminth therapy in wealthy developed countries is probably only effective in individual patients with specific genetic backgrounds and immunoregulatory deficits for which a particular helminth product happens to be relevant. This is how helminth products may be utilized in the future. However, until we know how to identify the appropriate combination of patient, genetics, disease, and helminth product, clinical trials may not yield useful results, and it is difficult to justify attempts to reconstruct the human biome with a helminth component.

## 5. Causes of failed microbial exposures; links to diet, SES and epidemiology

Table 2 lists some of the ways in which modern life-styles are reducing or distorting essential immunoregulatory exposures to the microbiota of mother, family and the natural environment. Many of them have been mentioned earlier in the text and will not be discussed in detail here. All the factors in the list alter microbial exposures and the microbiota, but clearly some of them are also detrimental to health in ways that are independent of effects on immunoregulation. For example, pollution alters the microbiota of the environment (186) and of the exposed public (103), but it is also directly toxic to humans. Smoking, while closely linked to low SES in many countries has profound effects on the microbiota (124), but is also directly toxic and carcinogenic. We need to know how much of the health deficit caused by smoking and pollution is due to distorted microbiota.

Similarly, diet has a major effect on the gut microbiota but it is increasingly suspected that some ingredients of ultraprocessed foods such as detergent-like emulsifiers and excitotoxins are also inherently toxic, causing a leaky gut and neuronal damage (118, 119). But poor diets can also fail to support immunoregulatory microbiota. For example an unvaried diet can reduce biodiversity of the microbiota (64, 117). Some modern diets associated with low SES can be frankly proinflammatory. There has been a disturbing increase in the incidence of cancers, notably breast and colorectal, appearing in people less than 50 years old (187). Recent studies suggest a role for exposures in early life (187), and the modern low SES Western diet is an obvious candidate. This diet is likely to be low in fibre (41) and SCFA (120) which are necessary for the establishment of immunoregulation as outlined in Section 3.3. Similarly, excessive consumption of sugars (including fructose) and artificial sweeteners leads to distortion of the microbiota, raised glycaemic responses, obesity and metabolic and cardiovascular problems (123–188). Such diets may also be deficient in micronutrients and vitamins, leading to potential deficiencies (12, 121).

These dietary factors, and others listed in Table 2, are closely associated with low SES in wealthy urban settings. In a classic study the difference in life expectancy in some parts of the British isles between wealthy and deprived areas was as high as 27 years (189). We and others have argued recently that much of this SES-associated health deficit might be secondary to inappropriate microbial exposures and inappropriate microbiota (190–192). We need to find out how much of the health deficit is mediated in this way because this might inspire new ways to combat the SES-linked health deficit.

### 5.1. SES and inconsistent epidemiology

Epidemiologists seeking links between low SES and allergic disorders should perhaps take note of the items in this list which can perhaps explain discordant results in this literature. In developing countries, low SES is often associated with subsistence agriculture in rural settings with abundant exposure to the natural environment. On the other hand, in wealthy urbanised countries low SES is associated with living in polluted urban slums with little access to green space while the wealthy have gardens and rural holiday homes. Similarly in developing countries access to Caesarean deliveries and antibiotics may be difficult for people of low SES, whereas in some rich countries, where these things are readily available, misuse of Caesarean deliveries and antibiotics can be more frequent amongst people of low SES, perhaps because they lack awareness of the disadvantages (193). These points can explain the enormous discrepancies between different epidemiological studies of allergic disorders which, for example, may or may not find links with low SES (194).

### 5.2. Epidemiology and multiple Treg subtypes

Treg biology provides another explanation for discordant epidemiology. There are several different subsets of Treg, which act at different stages in the development of an allergic response, including the initial expansion of T cell clones, polarisation towards Th2, regulation of IgE vs. IgG4 production and late effector pathways such as control of mast cell activation (195). Thus some patterns of Treg activation will block sensitisation, while other patterns of activation will fail to block sensitisation, but may still block clinical manifestations. This explains some instances of a lack of correlation between IgE or skin-prick test positivity and clinical manifestations of allergic disease (196). Different Treg subpopulations, located in different sites, might also explain the very variable correlation between skin, airway and gut manifestations of allergy (197, 198). We need more understanding of the role of different microbial exposures in expanding Treg cells that operate at each stage of the allergic response (195), and at different epithelial surfaces (198).

## 6. Conclusions

The Old Friends hypothesis emphasises the role of exposures to microorganisms with which humans co-evolved as essential drivers of the regulatory and anti-inflammatory arm of the immune system. Any hypothesis that seeks to explain the increases in allergic disorders must include an immunoregulatory component. Exposure to allergens and weakening of epithelial barriers certainly contribute (21), but they are unlikely to cause allergic responses unless there is also cell death providing the Th2-adjuvant effect of dsDNA (149–151), and unless defective immunoregulatory mechanisms permit it (17). Moreover, we need to explain the often simultaneous increases in other chronic inflammatory disorders such as autoimmunity and IBD (2, 4, 5) and systemic inflammatory states that predispose to cardiovascular, metabolic and psychiatric problems (6, 7, 199). Malfunctioning immunoregulation is likely to be fundamental to all these states, but most phenomena in biology are multifactorial. Things are most likely to happen when there are multiple reasons for them to do so. Indeed all the major hypotheses, whether they involve hygiene, “Old Friends”, biodiversity, novel allergens or leaky epithelium (Figure 2), can be regarded as different manifestations of a set of interacting gene-environment mismatches caused by modern lifestyles (200, 201).

We argue that mother, family and the natural environment (including the animals within it) provide the major microbial exposures that are needed to populate the gut microbiota and set up the immunoregulatory pathways that are defective in rich urban societies. The implication is that this mechanism is fundamental to all the current hypotheses. We now know that humans have indeed co-evolved with the microbiota, some members of which are becoming obligate symbionts (29, 30). We have minimal understanding of the physiological roles of these organisms, but we do know that at all body surfaces the microbiota drives development of both the effector and regulatory arms of the immune system (11–15). The biology of the multiple subtypes of Treg and the functional differences of Treg in different body sites can explain many of the wildly variable epidemiological findings on issues such as links to SES, correlations between different allergic disorders, and between sensitization and clinical manifestations (195, 198).

Infections also impact the immune system but mostly they provide data, specific immunity and non-specific activation of the innate immune system (“Trained Immunity”) rather than immunoregulation. Germ-line-encoded evolved dependence on infections is unlikely unless the infection was effectively inevitable throughout much of our evolution.

Finally, the Old Friends hypothesis points to many ways in which we can optimize exposures to the necessary microbial inputs. Almost all the detrimental factors listed in Table 2 can be offset by education, improving diets, minimizing use of antibiotics, reducing pollution, improving living conditions, designing better housing and providing access to green space.

## References

[1] Blackley CH. Experimental researches on the causes and nature of catarrhus aestivus (hay-fever and hay-asthma). London: Baillière Tindall and Cox (1873).

[2] Bach JF. The effect of infections on susceptibility to autoimmune and allergic diseases. N Engl J Med. (2002) 347(12):911–20. 10.1056/NEJMra020100 12239261

[3] Eder W Ege MJ von Mutius E. The asthma epidemic. N Engl J Med. (2006) 355(21):2226–35. 10.1056/NEJMra054308 17124020

[4] Stene LC Nafstad P. Relation between occurrence of type 1 diabetes and asthma. Lancet. (2001) 357:607. 10.1016/S0140-6736(00)04067-8 11558491

[5] Timm S Svanes C Janson C Sigsgaard T Johannessen A Gislason T Place of upbringing in early childhood as related to inflammatory bowel diseases in adulthood: a population-based cohort study in Northern Europe. Eur J Epidemiol. (2014) 29(6):429–37. 10.1007/s10654-014-9922-3 24916994 PMC4065648

[6] McDade TW. Early environments and the ecology of inflammation. Proc Natl Acad Sci U S A. (2012) 109(Suppl 2):17281–8. 10.1073/pnas.1202244109 23045646 PMC3477398

[7] Arnold N Lechner K Waldeyer C Shapiro MD Koenig W. Inflammation and cardiovascular disease: the future. Eur Cardiol. (2021) 16:e20. 10.15420/ecr.2020.50 34093741 PMC8157394

[8] Gimeno D Kivimaki M Brunner EJ Elovainio M De Vogli R Steptoe A Associations of C-reactive protein and interleukin-6 with cognitive symptoms of depression: 12-year follow-up of the Whitehall II study. Psychol Med. (2009) 39(3):413–23. 10.1017/S0033291708003723 18533059 PMC2788760

[9] Eraly SA Nievergelt CM Maihofer AX Barkauskas DA Biswas N Agorastos A Assessment of plasma C-reactive protein as a biomarker of posttraumatic stress disorder risk. JAMA Psychiatry. (2014) 71(4):423–31. 10.1001/jamapsychiatry.2013.4374 24576974 PMC4032578

[10] Khandaker GM Pearson RM Zammit S Lewis G Jones PB. Association of serum interleukin 6 and C-reactive protein in childhood with depression and psychosis in young adult life: a population-based longitudinal study. JAMA Psychiatry. (2014) 71:1121–8. 10.1001/jamapsychiatry.2014.1332 25133871 PMC4561502

[11] Pandiyan P Bhaskaran N Zou M Schneider E Jayaraman S Huehn J. Microbiome dependent regulation of Tregs and Th17 cells in mucosa. Front Immunol. (2019) 10:1–17. 10.3389/mmu.2019.00426 30906299 PMC6419713

[12] Yang W Cong Y. Gut microbiota-derived metabolites in the regulation of host immune responses and immune-related inflammatory diseases. Cell Mol Immunol. (2021) 18(4):866–77. 10.1038/s41423-021-00661-4 33707689 PMC8115644

[13] Atarashi K Tanoue T Shima T Imaoka A Kuwahara T Momose Y Induction of colonic regulatory T cells by indigenous Clostridium Species. Science. (2011) 331:337–41. 10.1126/science.1198469 21205640 PMC3969237

[14] Naik S Bouladoux N Linehan JL Han S Harrison OJ Wilhelm C Commensal-dendritic-cell interaction specifies a unique protective skin immune signature. Nature. (2015) 520:104–8. 10.1038/nature14052 25539086 PMC4667810

[15] Scharschmidt TC Vasquez KS Pauli ML Leitner EG Chu K Truong HA Commensal microbes and hair follicle morphogenesis coordinately drive treg migration into neonatal skin. Cell Host Microbe. (2017) 21(4):467–77.e5. 10.1016/j.chom.2017.03.001 28343820 PMC5516645

[16] Rook GAW Adams V Palmer R Brunet LR Hunt J Martinelli R. Mycobacteria and other environmental organisms as immunomodulators for immunoregulatory disorders. Springer Semin Immunopathol. (2004) 25(3–4):237–55. 10.1007/s00281-003-0148-9 15007629

[17] Rook GA. Regulation of the immune system by biodiversity from the natural environment: an ecosystem service essential to health. Proc Natl Acad Sci U S A. (2013) 110(46):18360–7. 10.1073/pnas.1313731110 24154724 PMC3831972

[18] Rook GAW. Darwinian medicine: we evolved to require continuing contact with the Microbiota of the natural environment. Evolution turns the inevitable into a necessity. In: Hurst CJ, editor. Microbes: The foundation stone of the biosphere. Cham, Switzerland: Springer International Publishing (2021). p. 327–64.

[19] von Hertzen L Hanski I Haahtela T. Natural immunity. Biodiversity loss and inflammatory diseases are two global megatrends that might be related. EMBO Rep. (2011) 12(11):1089–93. 10.1038/embor.2011.195 21979814 PMC3207110

[20] Florsheim EB Sullivan ZA Khoury-Hanold W Medzhitov R. Food allergy as a biological food quality control system. Cell. (2021) 184(6):1440–54. 10.1016/j.cell.2020.12.007 33450204

[21] Akdis CA. Does the epithelial barrier hypothesis explain the increase in allergy, autoimmunity and other chronic conditions? Nat Rev Immunol. (2021) 21(11):739–51. 10.1038/s41577-021-00538-7 33846604

[22] Rook GAW Bloomfield SF. Microbial exposures that establish immunoregulation are compatible with targeted hygiene. J Allergy Clin Immunol. (2021) 148(1):33–9. 10.1016/j.jaci.2021.05.008 34033844

[23] Imachi H Nobu MK Nakahara N Morono Y Ogawara M Takaki Y Isolation of an archaeon at the prokaryote–eukaryote interface. Nature. (2020) 577(7791):519–25. 10.1038/s41586-019-1916-6 31942073 PMC7015854

[24] Domazet-Loso T Tautz D. An ancient evolutionary origin of genes associated with human genetic diseases. Mol Biol Evol. (2008) 25(12):2699–707. 10.1093/molbev/msn214 18820252 PMC2582983

[25] Iyer LM Aravind L Coon SL Klein DC Koonin EV. Evolution of cell-cell signaling in animals: did late horizontal gene transfer from bacteria have a role? Trends Genet. (2004) 20(7):292–9. 10.1016/j.tig.2004.05.007 15219393

[26] Wikoff WR Anfora AT Liu J Schultz PG Lesley SA Peters EC Metabolomics analysis reveals large effects of gut microflora on mammalian blood metabolites. Proc Natl Acad Sci U S A. (2009) 106(10):3698–703. 10.1073/pnas.0812874106 19234110 PMC2656143

[27] Nakashima K Kimura S Ogawa Y Watanabe S Soma S Kaneko T Chitin-based barrier immunity and its loss predated mucus-colonization by indigenous gut microbiota. Nat Commun. (2018) 9(1):3402. 10.1038/s41467-018-05884-0 30143642 PMC6109156

[28] Flandroy L Poutahidis T Berg G Clarke G Dao M-C Decaestecker E The impact of human activities and lifestyles on the interlinked microbiota and health of humans and of ecosystems. Sci Total Environ. (2018) 627:1018–38. 10.1016/j.scitotenv.2018.01.288 29426121

[29] Moeller AH Caro-Quintero A Mjungu D Georgiev AV Lonsdorf EV Muller MN Cospeciation of gut microbiota with hominids. Science. (2016) 353(6297):380–2. 10.1126/science.aaf3951 27463672 PMC4995445

[30] Suzuki TA Fitzstevens JL Schmidt VT Enav H Huus KE Mbong Ngwese M Codiversification of gut microbiota with humans. Science. (2022) 377(6612):1328–32. 10.1126/science.abm7759 36108023 PMC10777373

[31] Rook GAW. Immune system. In: Brüne M Schiefenhövel W, editors. Oxford Handbook of evolutionary medicine. Oxford: Oxford University Press (2019). p. 411–61.

[32] Su LF Kidd BA Han A Kotzin JJ Davis MM. Virus-specific CD4(+) memory-phenotype T cells are abundant in unexposed adults. Immunity. (2013) 38(2):373–83. 10.1016/j.immuni.2012.10.021 23395677 PMC3626102

[33] Elias G Meysman P Bartholomeus E De Neuter N Keersmaekers N Suls A Preexisting memory CD4 T cells in naïve individuals confer robust immunity upon hepatitis B vaccination. eLife. (2022) 11:1–24. 10.7554/eLife.68388 PMC8824481 35074048

[34] Zegarra-Ruiz DF Kim DV Norwood K Kim M Wu W-JH Saldana-Morales FB Thymic development of gut-microbiota-specific T cells. Nature. (2021) 594:413–7. 10.1038/s41586-021-03531-1 33981034 PMC8323488

[35] Claesson MJ Jeffery IB Conde S Power SE O’Connor EM Cusack S Gut microbiota composition correlates with diet and health in the elderly. Nature. (2012) 488(7410):178–84. 10.1038/nature11319 22797518

[36] Narushima S Sugiura Y Oshima K Atarashi K Hattori M Suematsu M Characterization of the 17 strains of regulatory T cell-inducing human-derived Clostridia. Gut Microbes. (2014) 5(3):333–9. 10.4161/gmic.28572 24642476 PMC4153770

[37] Dalile B Van Oudenhove L Vervliet B Verbeke K. The role of short-chain fatty acids in microbiota–gut–brain communication. Nat Rev Gastroenterol Hepatol. (2019) 16(8):461–78. 10.1038/s41575-019-0157-3 31123355

[38] Cekanaviciute E Pröbstel A-K Thomann A Runia TF Casaccia P Katz Sand I Multiple sclerosis-associated changes in the composition and immune functions of spore-forming bacteria. mSystems. (2018) 3(6):1–12. 10.1128/mSystems.00083-18 PMC6222044 30417113

[39] Hiemstra PS Amatngalim GD van der Does AM Taube C. Antimicrobial peptides and innate lung defenses: role in infectious and noninfectious lung diseases and therapeutic applications. Chest. (2016) 149(2):545–51. 10.1378/chest.15-1353 26502035

[40] Schulz O Pabst O. Antigen sampling in the small intestine. Trends Immunol. (2013) 34(4):155–61. 10.1016/j.it.2012.09.006 23083727

[41] Tan J McKenzie C Vuillermin PJ Goverse G Vinuesa CG Mebius RE Dietary fiber and bacterial SCFA enhance oral tolerance and protect against food allergy through diverse cellular pathways. Cell Rep. (2016) 15(12):2809–24. 10.1016/j.celrep.2016.05.047 27332875

[42] Xiong R-G Zhou D-D Wu S-X Huang S-Y Saimaiti A Yang Z-J Health benefits and side effects of short-chain fatty acids. Foods. (2022) 11(18):1–24. 10.3390/foods11182863 PMC9498509 36140990

[43] Fiore A Murray PJ. Tryptophan and indole metabolism in immune regulation. Curr Opin Immunol. (2021) 70:7–14. 10.1016/j.coi.2020.12.001 33418116

[44] Hang S Paik D Yao L Kim E Trinath J Lu J Bile acid metabolites control TH17 and Treg cell differentiation. Nature. (2019) 576(7785):143–8. 10.1038/s41586-019-1785-z 31776512 PMC6949019

[45] Ikeda K Kinoshita M Kayama H Nagamori S Kongpracha P Umemoto E Slc3a2 mediates branched-chain amino-acid-dependent maintenance of regulatory T cells. Cell Rep. (2017) 21(7):1824–38. 10.1016/j.celrep.2017.10.082 29141216

[46] Stein MM Hrusch CL Gozdz J Igartua C Pivniouk V Murray SE Innate immunity and asthma risk in amish and hutterite farm children. N Engl J Med. (2016) 375(5):411–21. 10.1056/NEJMoa1508749 27518660 PMC5137793

[47] Hrusch CL Stein MM Gozdz J Holbreich M von Mutius E Vercelli D T-cell phenotypes are associated with serum IgE levels in Amish and Hutterite children. J Allergy Clin Immunol. (2019) 144(5):1391–401.e10. 10.1016/j.jaci.2019.07.034 31401285 PMC6842432

[48] Moura-Alves P Faé K Houthuys E Dorhoi A Kreuchwig A Furkert J Ahr sensing of bacterial pigments regulates antibacterial defence. Nature. (2014) 512(7515):387–92. 10.1038/nature13684 25119038

[49] Moore MN. Do airborne biogenic chemicals interact with the PI3K/Akt/mTOR cell signalling pathway to benefit human health and wellbeing in rural and coastal environments? Environ Res. (2015) 140:65–75. 10.1016/j.envres.2015.03.015 25825132

[50] Hehemann JH Correc G Barbeyron T Helbert W Czjzek M Michel G. Transfer of carbohydrate-active enzymes from marine bacteria to Japanese gut microbiota. Nature. (2010) 464(7290):908–12. 10.1038/nature08937 20376150

[51] Smillie CS Smith MB Friedman J Cordero OX David LA Alm EJ. Ecology drives a global network of gene exchange connecting the human microbiome. Nature. (2011) 480(7376):241–4. 10.1038/nature10571 22037308

[52] Yaffe E Relman DA. Tracking microbial evolution in the human gut using Hi-C reveals extensive horizontal gene transfer, persistence and adaptation. Nat Microbiol. (2019) 5:343–53. 10.1038/s41564-019-0625-0 31873203 PMC6992475

[53] Díaz-Garrido N Badia J Baldomà L. Microbiota-derived extracellular vesicles in interkingdom communication in the gut. J Extracell Vesicles. (2021) 10(13):e12161. 10.1002/jev2.12161 34738337 PMC8568775

[54] Aaby P Bhuiya A Nahar L Knudsen K de Francisco A Strong M. The survival benefit of measles immunization may not be explained entirely by the prevention of measles disease: a community study from rural Bangladesh. Int J Epidemiol. (2003) 32(1):106–15. 10.1093/ije/dyg005 12690020

[55] Barton ES White DW Cathelyn JS Brett-McClellan KA Engle M Diamond MS Herpesvirus latency confers symbiotic protection from bacterial infection. Nature. (2007) 447(7142):326–9. 10.1038/nature05762 17507983

[56] Chen Y Blaser MJ. Helicobacter pylori colonization is inversely associated with childhood asthma. J Infect Dis. (2008) 198(4):553–60. 10.1086/590158 18598192 PMC3902975

[57] Smieja MJ Marchetti CA Cook DC Smaill FM. Isoniazid for preventing tuberculosis in non-HIV infected persons. Cochrane Database Syst Rev). (1999) 1999(2):Cd001363. 10.1002/14651858.cd001363 PMC6532737 10796642

[58] Benn CS Fisker AB Rieckmann A Sørup S Aaby P. Vaccinology: time to change the paradigm? Lancet Infect Dis. (2020) 20(10):e274–83. 10.1016/s1473-3099(19)30742-x 32645296

[59] Aaby P Benn CS Flanagan KL Klein SL Kollmann TR Lynn DJ The non-specific and sex-differential effects of vaccines. Nat Rev Immunol. (2020) 20(8):464–70. 10.1038/s41577-020-0338-x 32461674 PMC7252419

[60] Netea MG Schlitzer A Placek K Joosten LAB Schultze JL. Innate and adaptive immune memory: an evolutionary continuum in the host’s response to pathogens. Cell Host Microbe. (2019) 25(1):13–26. 10.1016/j.chom.2018.12.006 30629914

[61] Van Belleghem JD Dabrowska K Vaneechoutte M Barr JJ Bollyky PL. Interactions between bacteriophage, bacteria, and the mammalian immune system. Viruses. (2018) 11(1):1–22. 10.3390/v11010010 30585199 PMC6356784

[62] Ott SJ Waetzig GH Rehman A Moltzau-Anderson J Bharti R Grasis JA Efficacy of sterile fecal filtrate transfer for treating patients with Clostridium difficile infection. Gastroenterology. (2017) 152(4):799–811.e7. 10.1053/j.gastro.2016.11.010 27866880

[63] Sinha A Maurice CF. Bacteriophages: uncharacterized and dynamic regulators of the immune system. Mediators Inflamm. (2019) 2019:3730519. 10.1155/2019/3730519 31582898 PMC6754933

[64] Biagi E Franceschi C Rampelli S Severgnini M Ostan R Turroni S Gut microbiota and extreme longevity. Curr Biol. (2016) 26(11):1480–5. 10.1016/j.cub.2016.04.016 27185560

[65] Vangay P Johnson AJ Ward TL Al-Ghalith GA Shields-Cutler RR Hillmann BM US immigration westernizes the human gut microbiome. Cell. (2018) 175(4):962–72.e10. 10.1016/j.cell.2018.10.029 30388453 PMC6498444

[66] Hill JH Franzosa EA Huttenhower C Guillemin K. A conserved bacterial protein induces pancreatic beta cell expansion during zebrafish development. eLife. (2016) 5:e20145. 10.7554/eLife.20145 27960075 PMC5154760

[67] de Mazancourt C Johnson E Barraclough TG. Biodiversity inhibits species’ evolutionary responses to changing environments. Ecol Lett. (2008) 11(4):380–8. 10.1111/j.1461-0248.2008.01152.x 18248449

[68] Tsui C Kong EF Jabra-Rizk MA Mobley H. Pathogenesis of Candida albicans biofilm. Pathog Dis. (2016) 74(4):ftw018. 10.1093/femspd/ftw018 26960943 PMC5975230

[69] Buret AG Motta J-P Allain T Ferraz J Wallace JL. Pathobiont release from dysbiotic gut microbiota biofilms in intestinal inflammatory diseases: a role for iron? J Biomed Sci. (2019) 26(1):1. 10.1186/s12929-018-0495-4 30602371 PMC6317250

[70] Coppola S Avagliano C Sacchi A Laneri S Calignano A Voto L Potential clinical applications of the postbiotic butyrate in human skin diseases. Molecules (Basel, Switzerland). (2022) 27(6):1849. 10.3390/molecules27061849 35335213 PMC8949901

[71] Whitlock DR Feelisch M. Soil bacteria, nitrite, and the skin. In: Rook GAW, editor. The hygiene hypothesis and darwinian medicine. Progress in inflammation research. Basel: Birkhäuser (2009). p. 103–16.

[72] Maura D Elmekki N Goddard CA. The ammonia oxidizing bacterium Nitrosomonas eutropha blocks T helper 2 cell polarization via the anti-inflammatory cytokine IL-10. Sci Rep. (2021) 11(1):14162. 10.1038/s41598-021-93299-1 34238943 PMC8266879

[73] Lee NY Ibrahim O Khetarpal S Gaber M Jamas S Gryllos I Dermal microflora restoration with ammonia-oxidizing bacteria nitrosomonas Eutropha in the treatment of keratosis pilaris: a randomized clinical trial. J Drugs Dermatol. (2018) 17(3):285–8. PMID: 29537446

[74] Alves-Rosa F Vulcano M Beigier-Bompadre M Fernández G Palermo M Isturiz MA. Interleukin-1beta induces in vivo tolerance to lipopolysaccharide in mice. Clin Exp Immunol. (2002) 128(2):221–8. 10.1046/j.1365-2249.2002.01828.x 12041508 PMC1906386

[75] Jia L Lu J Zhou Y Tao Y Xu H Zheng W Tolerogenic dendritic cells induced the enrichment of CD4+Foxp3+ regulatory T cells via TGF-β in mesenteric lymph nodes of murine LPS-induced tolerance model. Clin Immunol. (2018) 197:118–29. 10.1016/j.clim.2018.09.010 30248398

[76] Schuijs MJ Willart MA Vergote K Gras D Deswarte K Ege MJ Farm dust and endotoxin protect against allergy through A20 induction in lung epithelial cells. Science. (2015) 349(6252):1106–10. 10.1126/science.aac6623 26339029

[77] Anstadt EJ Fujiwara M Wasko N Nichols F Clark RB. TLR tolerance as a treatment for central nervous system autoimmunity. J Immunol. (2016) 197(6):2110. 10.4049/jimmunol.1600876 27503211

[78] Krieg AM Wu T Weeratna R Efler SM Love-Homan L Yang L Sequence motifs in adenoviral DNA block immune activation by stimulatory CpG motifs. Proc Natl Acad Sci U S A. (1998) 95(21):12631–6. 10.1073/pnas.95.21.12631 9770537 PMC22882

[79] Hiramatsu Y Satho T Hyakutake M Irie K Mishima K Miake F The anti-inflammatory effects of a high-frequency oligodeoxynucleotide from the genomic DNA of Lactobacillus casei. Int Immunopharmacol. (2014) 23(1):139–47. 10.1016/j.intimp.2014.08.013 25193776

[80] Mazhary Z Allahyari Fard N Minuchehr Z Javanshir N. Package of anti-allergic probiotic Lactobacillus by focusing on the regulatory role of immunosuppressive motifs in allergy. Inf Med Unlocked. (2020) 18:100280. 10.1016/j.imu.2019.100280

[81] Rachmilewitz D Katakura K Karmeli F Hayashi T Reinus C Rudensky B Toll-like receptor 9 signaling mediates the anti-inflammatory effects of probiotics in murine experimental colitis. Gastroenterology. (2004) 126(2):520–8. 10.1053/j.gastro.2003.11.019 14762789

[82] Liu S da Cunha AP Rezende RM Cialic R Wei Z Bry L The host shapes the gut microbiota via fecal MicroRNA. Cell Host Microbe. (2016) 19(1):32–43. 10.1016/j.chom.2015.12.005 26764595 PMC4847146

[83] Gusareva ES Acerbi E Lau KJX Luhung I Premkrishnan BNV Kolundžija S Microbial communities in the tropical air ecosystem follow a precise diel cycle. Proc Natl Acad Sci USA. (2019) 116(46):23299. 10.1073/pnas.1908493116 31659049 PMC6859341

[84] Mohammadi-Bardbori A Bengtsson J Rannug U Rannug A Wincent E. Quercetin, resveratrol, and curcumin are indirect activators of the aryl hydrocarbon receptor (AHR). Chem Res Toxicol. (2012) 25(9):1878–84. 10.1021/tx300169e 22867086

[85] Strachan DP. Hay fever, hygiene, and household size. Brit Med J. (1989) 299(6710):1259–60. 10.1136/bmj.299.6710.1259 2513902 PMC1838109

[86] Bartlett MS. Measles periodicity and community size. J Royal Stat Soc Series A (General). (1957) 120(1):48–70. 10.2307/2342553

[87] Black FL. Measles endemicity in insular populations: critical community size and its evolutionary implication. J Theor Biol. (1966) 11(2):207–11. 10.0022-5193(66)90161-5 5965486 10.1016/0022-5193(66)90161-5

[88] Furuse Y Suzuki A Oshitani H. Origin of measles virus: divergence from rinderpest virus between the 11th and 12th centuries. Virol J. (2010) 7:52. 10.1186/1743-422X-7-52 20202190 PMC2838858

[89] Benn CS Melbye M Wohlfahrt J Bjorksten B Aaby P. Cohort study of sibling effect, infectious diseases, and risk of atopic dermatitis during first 18 months of life. Brit Med J. (2004) 328:1223–8. 10.1136/bmj.38069.512245.FE 15121716 PMC416593

[90] Bremner SA Carey IM DeWilde S Richards N Maier WC Hilton SR Infections presenting for clinical care in early life and later risk of hay fever in two UK birth cohorts. Allergy. (2008) 63(3):274–83. 10.1111/j.1398-9995.2007.01599.x 18269673

[91] Dunder T Tapiainen T Pokka T Uhari M. Infections in child day care centers and later development of asthma, allergic rhinitis, and atopic dermatitis: prospective follow-up survey 12 years after controlled randomized hygiene intervention. Arch Pediatr Adolesc Med. (2007) 161(10):972–7. 10.1001/archpedi.161.10.972 17909141

[92] Johnston SL Pattemore PK Sanderson G Smith S Lampe F Josephs L Community study of role of viral infections in exacerbations of asthma in 9–11 year old children. Br Med J. (1995) 310(6989):1225. 10.1136/bmj.310.6989.1225 7767192 PMC2549614

[93] Jartti T Gern JE. Role of viral infections in the development and exacerbation of asthma in children. J Allergy Clin Immunol. (2017) 140(4):895–906. 10.1016/j.jaci.2017.08.003 28987219 PMC7172811

[94] Galazzo G van Best N Bervoets L Dapaah IO Savelkoul PH Hornef MW Development of the microbiota and associations with birth mode, diet, and atopic disorders in a longitudinal analysis of stool samples, collected from infancy through early childhood. Gastroenterology. (2020) 158(6):1584–96. 10.1053/j.gastro.2020.01.024 31958431

[95] Renz H Skevaki C. Early life microbial exposures and allergy risks: opportunities for prevention. Nat Rev Immunol. (2020) 21:177–91. 10.1038/s41577-020-00420-y 32918062

[96] Pullinger EJ. The influence of Tuberculosis upon the development of Brucella abortus infection. J Hyg (Lond). (1936) 36(3):456–66. 10.1017/s0022172400043783 20475342 PMC2171002

[97] Elberg SS Schneider P Fong J. Cross-immunity between Brucella melitensis and Mycobacterium tuberculosis; intracellular behavior of Brucella melitensis in monocytes from vaccinated animals. J Exp Med. (1957) 106(4):545–54. 10.1084/jem.106.4.545 13475612 PMC2136813

[98] Gregorio SB Maasab HF Eveland WC. Interaction of Listeria monocytogenes and influenza in an animal model. Health Lab Sci. (1976) 13(4):250–7. 10263

[99] Netea MG Joosten LAB Latz E Mills KHG Natoli G Stunnenberg HG Trained immunity: a program of innate immune memory in health and disease. Science. (2016) 352(6284):aaf1098. 10.1126/science.aaf1098 27102489 PMC5087274

[100] Murphy DM Mills KHG Basdeo SA. The effects of trained innate immunity on T cell responses; clinical implications and knowledge gaps for future research. Front Immunol. (2021) 12:1–12. 10.3389/fimmu.2021.706583 PMC8417102 34489958

[101] Hesselmar B Sjoberg F Saalman R Aberg N Adlerberth I Wold AE. Pacifier cleaning practices and risk of allergy development. Pediatrics. (2013) 131(6):e1829–37. 10.1542/peds.2012-3345 23650304

[102] Alderete TL Jones RB Chen Z Kim JS Habre R Lurmann F Exposure to traffic-related air pollution and the composition of the gut microbiota in overweight and obese adolescents. Environ Res. (2018) 161:472–8. 10.1016/j.envres.2017.11.046 29220800 PMC5747978

[103] Fouladi F Bailey MJ Patterson WB Sioda M Blakley IC Fodor AA Air pollution exposure is associated with the gut microbiome as revealed by shotgun metagenomic sequencing. Environ Int. (2020) 138:105604. 10.1016/j.envint.2020.105604 32135388 PMC7181344

[104] Andersson MA Mikkola R Kroppenstedt RM Rainey FA Peltola J Helin J The mitochondrial toxin produced by Streptomyces griseus strains isolated from an indoor environment is valinomycin. Appl Environ Microbiol. (1998) 64(12):4767–73. 10.1128/AEM.64.12.4767-4773.1998 9835560 PMC90920

[105] Sahlberg B Wieslander G Norback D. Sick building syndrome in relation to domestic exposure in Sweden--a cohort study from 1991 to 2001. Scand J Public Health. (2010) 38(3):232–8. 10.1177/1403494809350517 19850651

[106] Hyvärinen A Meklin T Vepsäläinen A Nevalainen A. Fungi and actinobacteria in moisture-damaged building materials — concentrations and diversity. Int Biodeterior Biodegrad. (2002) 49(1):27–37. 10.1016/S0964-8305(01)00103-2

[107] Ingham T Keall M Jones B Aldridge DRT Dowell AC Davies C Damp mouldy housing and early childhood hospital admissions for acute respiratory infection: a case control study. Thorax. (2019) 74(9):849–57. 10.1136/thoraxjnl-2018-212979 31413146 PMC6824607

[108] Nesbitt L Meitner MJ Girling C Sheppard SRJ Lu Y. Who has access to urban vegetation? A spatial analysis of distributional green equity in 10 US cities. Landsc Urban Plann. (2019) 181:51–79. 10.1016/j.landurbplan.2018.08.007

[109] Roslund MI Puhakka R Grönroos M Nurminen N Oikarinen S Gazali AM Biodiversity intervention enhances immune regulation and health-associated commensal microbiota among daycare children. Sci Adv. (2020) 6(42):eaba2578. 10.1126/sciadv.aba2578 33055153 PMC7556828

[110] Engemann K Pedersen CB Arge L Tsirogiannis C Mortensen PB Svenning J-C. Residential green space in childhood is associated with lower risk of psychiatric disorders from adolescence into adulthood. Proc Natl Acad Sci USA. (2019) 116(11):5188–93. 10.1073/pnas.1807504116 30804178 PMC6421415

[111] Mitchell R Popham F. Effect of exposure to natural environment on health inequalities: an observational population study. Lancet. (2008) 372(9650):1655–60. 10.1016/S0140-6736(08)61689-X 18994663

[112] Bosman ES Albert AY Lui H Dutz JP Vallance BA. Skin exposure to narrow band ultraviolet (UVB) light modulates the human intestinal microbiome. Front Microbiol. (2019) 10:2410. 10.3389/fmicb.2019.02410 31708890 PMC6821880

[113] Yamamoto EA Jørgensen TN. Relationships between vitamin D, gut microbiome, and systemic autoimmunity. Front Immunol. (2020) 10:3141. 10.3389/fimmu.2019.03141 32038645 PMC6985452

[114] Smith RP Easson C Lyle SM Kapoor R Donnelly CP Davidson EJ Gut microbiome diversity is associated with sleep physiology in humans. PLoS ONE. (2019) 14(10):e0222394. 10.1371/journal.pone.0222394 31589627 PMC6779243

[115] Madison A Kiecolt-Glaser JK. Stress, depression, diet, and the gut microbiota: human–bacteria interactions at the core of psychoneuroimmunology and nutrition. Curr Opin Behav Sci. (2019) 28:105–10. 10.1016/j.cobeha.2019.01.011 32395568 PMC7213601

[116] Cai Y Zijlema WL Sørgjerd EP Doiron D de Hoogh K Hodgson S Impact of road traffic noise on obesity measures: observational study of three European cohorts. Environ Res. (2020) 191:110013. 10.1016/j.envres.2020.110013 32805247

[117] Makki K Deehan EC Walter J Bäckhed F. The impact of dietary fiber on gut Microbiota in host health and disease. Cell Host Microbe. (2018) 23(6):705–15. 10.1016/j.chom.2018.05.012 29902436

[118] Murray SL Holton KF. Effects of a diet low in excitotoxins on PTSD symptoms and related biomarkers. Nutr Neurosci. (2022):1–11. 10.1080/1028415X.2022.2152932 36484432

[119] Chassaing B Koren O Goodrich JK Poole AC Srinivasan S Ley RE Dietary emulsifiers impact the mouse gut microbiota promoting colitis and metabolic syndrome. Nature. (2015) 519(7541):92–6. 10.1038/nature14232 25731162 PMC4910713

[120] van der Hee B Wells JM. Microbial regulation of host physiology by short-chain fatty acids. Trends Microbiol. (2021) 29(8):700–12. 10.1016/j.tim.2021.02.001 33674141

[121] Yoshii K Hosomi K Sawane K Kunisawa J. Metabolism of dietary and microbial vitamin B family in the regulation of host immunity. Front Nutr. (2019) 6:48. 10.3389/fnut.2019.00048 31058161 PMC6478888

[122] Hartstra AV Bouter KE Backhed F Nieuwdorp M. Insights into the role of the microbiome in obesity and type 2 diabetes. Diabetes Care. (2015) 38(1):159–65. 10.2337/dc14-0769 25538312

[123] Suez J Korem T Zilberman-Schapira G Segal E Elinav E. Non-caloric artificial sweeteners and the microbiome: findings and challenges. Gut Microbes. (2015) 6(2):149–55. 10.1080/19490976.2015.1017700 25831243 PMC4615743

[124] Huang C Shi G. Smoking and microbiome in oral, airway, gut and some systemic diseases. J Transl Med. (2019) 17(1):225. 10.1186/s12967-019-1971-7 31307469 PMC6632217

[125] Korpela K Salonen A Virta LJ Kekkonen RA Forslund K Bork P Intestinal microbiome is related to lifetime antibiotic use in Finnish pre-school children. Nat Commun. (2016) 7:10410. 10.1038/ncomms10410 26811868 PMC4737757

[126] Shao X Ding X Wang B Li L An X Yao Q Antibiotic exposure in early life increases risk of childhood obesity: a systematic review and meta-analysis. Front Endocrinol (Lausanne). (2017) 8:170. 10.3389/fendo.2017.00170 28775712 PMC5517403

[127] Metzler S Frei R Schmaußer-Hechfellner E von Mutius E Pekkanen J Karvonen AM Association between antibiotic treatment during pregnancy and infancy and the development of allergic diseases. Pediatr Allergy Immunol. (2019) 30(4):423–33. 10.1111/pai.13039 30734960

[128] Adlercreutz H Martin F Pulkkinen M Dencker H Rimer U Sjoberg NO Intestinal metabolism of estrogens. J Clin Endocrinol Metab. (1976) 43(3):497–505. 10.1210/jcem-43-3-497 956337

[129] Rook GAW. Human evolution, microorganisms, socioeconomic status and reconciling necessary microbial exposures with essential hygiene. In: Rook GAW Lowry CA, editors. Evolution, biodiversity and a reassessment of the hygiene hypothesis. Progress in inflammation research. Vol. 89. Cham, Switzerland: Springer Nature Switzerland AG (2022). p. 27–66.

[130] Bertoncello C Ferro A Fonzo M Zanovello S Napoletano G Russo F Socioeconomic determinants in vaccine hesitancy and vaccine refusal in Italy. Vaccines (Basel). (2020) 8(2):276. 10.3390/vaccines8020276 32516936 PMC7349972

[131] Roswall J Olsson LM Kovatcheva-Datchary P Nilsson S Tremaroli V Simon MC Developmental trajectory of the healthy human gut microbiota during the first 5 years of life. Cell Host Microbe. (2021) 29:765–76.e3. 10.1016/j.chom.2021.02.021 33794185

[132] Johnson KVA. Gut microbiome composition and diversity are related to human personality traits. Hum Microb J. (2020) 15:100069. 10.1016/j.humic.2019.100069 PMC8336012 34435164

[133] Brito IL Gurry T Zhao S Huang K Young SK Shea TP Transmission of human-associated microbiota along family and social networks. Nat Microbiol. (2019) 4(6):964–71. 10.1038/s41564-019-0409-6 30911128 PMC7450247

[134] Adams RI Bhangar S Dannemiller KC Eisen JA Fierer N Gilbert JA Ten questions concerning the microbiomes of buildings. Build Environ. (2016) 109:224–34. 10.1016/j.buildenv.2016.09.001

[135] McCall L-I Callewaert C Zhu Q Song SJ Bouslimani A Minich JJ Home chemical and microbial transitions across urbanization. Nat Microbiol. (2019) 5:108–15. 10.1038/s41564-019-0593-4 31686026 PMC7895447

[136] Parajuli A Gronroos M Siter N Puhakka R Vari HK Roslund MI Urbanization reduces transfer of diverse environmental microbiota indoors. Front Microbiol. (2018) 9:84. 10.3389/fmicb.2018.00084 29467728 PMC5808279

[137] Salo MJ Marik T Mikkola R Andersson MA Kredics L Salonen H Penicillium expansum strain isolated from indoor building material was able to grow on gypsum board and emitted guttation droplets containing chaetoglobosins and communesins A, B and D. J Appl Microbiol. (2019) 127(4):1135–47. 10.1111/jam.14369 31271686 PMC6852191

[138] Ege MJ Mayer M Normand A-C Genuneit J Cookson WOCM Braun-Fahrländer C Exposure to environmental microorganisms and childhood asthma. N Engl J Med. (2011) 364(8):701–9. 10.1056/NEJMoa1007302 21345099

[139] Hesselmar B Hicke-Roberts A Lundell AC Adlerberth I Rudin A Saalman R Pet-keeping in early life reduces the risk of allergy in a dose-dependent fashion. PLoS One. (2018) 13(12):e0208472. 10.1371/journal.pone.0208472 30566481 PMC6300190

[140] Kirjavainen PV Karvonen AM Adams RI Taubel M Roponen M Tuoresmaki P Farm-like indoor microbiota in non-farm homes protects children from asthma development. Nat Med. (2019) 25(7):1089–95. 10.1038/s41591-019-0469-4 31209334 PMC7617062

[141] Stoppard M. Fighting the germ of our near obsessive cleanliness. Mumbai Mirror. (2015). Available at: https://mumbaimirror.indiatimes.com/others/health-lifestyle/Fighting-the-germ-of-our-near-obsessive-cleanliness/articleshow/50085504.cms

[142] Sherriff A Golding J, ALSPAC Study Team. Hygiene levels in a contemporary population cohort are associated with wheezing and atopic eczema in preschool infants. Arch Dis Child. (2002) 87(1):26–9. 10.1136/adc.87.1.26 12089117 PMC1751124

[143] Weber J Illi S Nowak D Schierl R Holst O von Mutius E Asthma and the hygiene hypothesis. Does cleanliness matter? Am J Respir Crit Care Med. (2015) 191(5):522–9. 10.1164/rccm.201410-1899OC 25584716

[144] Lemire P Dumas O Chanoine S Temam S Severi G Boutron-Ruault M-C Domestic exposure to irritant cleaning agents and asthma in women. Environ Int. (2020) 144:106017. 10.1016/j.envint.2020.106017 32829252

[145] Gallucci S Matzinger P. Danger signals: SOS to the immune system. Curr Opin Immunol. (2001) 13(1):114–9. 10.1016/s0952-7915(00)00191-6 11154927

[146] Sasaki E Asanuma H Momose H Furuhata K Mizukami T Hamaguchi I. Immunogenicity and toxicity of different adjuvants can be characterized by profiling lung biomarker genes after nasal immunization. Front Immunol. (2020) 11:2171. 10.3389/fimmu.2020.02171 33013912 PMC7516075

[147] Sherriff A Golding J, ALSPAC Study Team.. Factors associated with different hygiene practices in the homes of 15 month old infants. Arch Dis Child. (2002) 87(1):30–5. 10.1136/adc.87.1.30 12089118 PMC1751130

[148] McKee AS Burchill MA Munks MW Jin L Kappler JW Friedman RS Host DNA released in response to aluminum adjuvant enhances MHC class II-mediated antigen presentation and prolongs CD4 T-cell interactions with dendritic cells. Proc Natl Acad Sci USA. (2013) 110(12):E1122–31. 10.1073/pnas.1300392110 23447566 PMC3607057

[149] Ma R Ortiz Serrano TP Davis J Prigge AD Ridge KM. The cGAS-STING pathway: the role of self-DNA sensing in inflammatory lung disease. FASEB J. (2020) 34(10):13156–70. 10.1096/fj.202001607R 32860267 PMC8121456

[150] Han Y Chen L Liu H Jin Z Wu Y Wu Y Airway epithelial cGAS is critical for induction of experimental allergic airway inflammation. J Immunol. (2020) 204(6):1437–47. 10.4049/jimmunol.1900869 32034061

[151] Rook G. Does exposing infants to cleaning agents containing detergents and substances causing cell death (and release of dsDNA) predispose to allergic disorders? (2023). Available at: https://www.grahamrook.net/cleaning/ (Cited 10 April 2023).

[152] von Mutius E Vercelli D. Farm living: effects on childhood asthma and allergy. Nat Rev Immunol. (2010) 10(12):861–8. 10.1038/nri2871 21060319

[153] Okabe H Hashimoto K Yamada M Ono T Yaginuma K Kume Y Associations between fetal or infancy pet exposure and food allergies: the Japan environment and children’s study. PLoS One. (2023) 18(3):e0282725. 10.1371/journal.pone.0282725 36989214 PMC10057762

[154] Panzer AR Sitarik AR Fadrosh D Havstad SL Jones K Davidson B The impact of prenatal dog keeping on infant gut microbiota development. Clin Exp Allergy. (2023) 53:833–45. 10.1111/cea.14303 36916778 PMC11163251

[155] Hanski I von Hertzen L Fyhrquist N Koskinen K Torppa K Laatikainen T Environmental biodiversity, human microbiota, and allergy are interrelated. Proc Natl Acad Sci U S A. (2012) 109(21):8334–9. 10.1073/pnas.1205624109 22566627 PMC3361383

[156] Elten M Benchimol EI Fell DB Kuenzig ME Smith G Kaplan GG Residential greenspace in childhood reduces risk of pediatric inflammatory bowel disease: a population-based cohort study. Am J Gastroenterol. (2021) 116(2):347–53. 10.14309/ajg.0000000000000990 33038129

[157] Joung YS Ge Z Buie CR. Bioaerosol generation by raindrops on soil. Nat Commun. (2017) 8:14668. 10.1038/ncomms14668 28267145 PMC5344306

[158] Troyer K. Behavioral acquisition of the hindgut fermentation system by hatchling Iguana iguana. Behav Ecol Sociobiol. (1984) 14(3):189–93. 10.1007/BF00299618

[159] Krishnamani R Mahaney WC. Geophagy among primates: adaptive significance and ecological consequences. Anim Behav. (2000) 59(5):899–915. 10.1006/anbe.1999.1376 10860518

[160] Sing D Sing CF. Impact of direct soil exposures from airborne dust and geophagy on human health. Int J Environ Res Public Health. (2010) 7(3):1205–23. 10.3390/ijerph7031205 20617027 PMC2872320

[161] Geissler PW Mwaniki DL Thiong’o F Friis H. Geophagy among school children in Western Kenya. Trop Med Int Health. (1997) 2(7):624–30. 10.1046/j.1365-3156.1997.d01-345.x 9270730

[162] Brame JE Liddicoat C Abbott CA Breed MF. The potential of outdoor environments to supply beneficial butyrate-producing bacteria to humans. Sci Total Environ. (2021) 777:146063. 10.1016/j.scitotenv.2021.146063 33684759

[163] Zhao G Vatanen T Droit L Park A Kostic AD Poon TW Intestinal virome changes precede autoimmunity in type I diabetes-susceptible children. Proc Natl Acad Sci U S A. (2017) 114(30):E6166–75. 10.1073/pnas.1706359114 28696303 PMC5544325

[164] Nicholson WL. Roles of Bacillus endospores in the environment. Cell Mol Life Sci. (2002) 59(3):410–6. 10.1007/s00018-002-8433-7 11964119 PMC11337551

[165] Browne HP Forster SC Anonye BO Kumar N Neville BA Stares MD Culturing of “unculturable” human microbiota reveals novel taxa and extensive sporulation. Nature. (2016) 533(7604):543–6. 10.1038/nature17645 27144353 PMC4890681

[166] Egan M Dempsey E Ryan CA Ross RP Stanton C. The sporobiota of the human gut. Gut Microbes. (2021) 13(1):1–17. 10.1080/19490976.2020.1863134 PMC7801112 33406976

[167] Nocera AL Mueller SK Stephan JR Hing L Seifert P Han X Exosome swarms eliminate airway pathogens and provide passive epithelial immunoprotection through nitric oxide. J Allergy Clin Immunol. (2019) 143(4):1525–35 e1. 10.1016/j.jaci.2018.08.046 30442371

[168] Adams K Weber KS Johnson SM. Exposome and immunity training: how pathogen exposure order influences innate immune cell lineage commitment and function. Int J Mol Sci. (2020) 21(22):8462. 10.3390/ijms21228462 33187101 PMC7697998

[169] Greaves M. A causal mechanism for childhood acute lymphoblastic leukaemia. Nat Rev Cancer. (2018) 18(8):471–84. 10.1038/s41568-018-0015-6 29784935 PMC6986894

[170] Harrison LC Perrett KP Jachno K Nolan TM Honeyman MC. Does rotavirus turn on type 1 diabetes? PLoS Pathog. (2019) 15(10):e1007965. 10.1371/journal.ppat.1007965 31600345 PMC6786515

[171] Matricardi PM Rosmini F Panetta V Ferrigno L Bonini S. Hay fever and asthma in relation to markers of infection in the United States. J Allergy Clin Immunol. (2002) 110(3):381–7. 10.1067/mai.2002.126658 12209083

[172] Kyburz A Fallegger A Zhang X Altobelli A Artola-Boran M Borbet T Transmaternal Helicobacter pylori exposure reduces allergic airway inflammation in offspring through regulatory T cells. J Allergy Clin Immunol. (2019) 143(4):1496–512.e11. 10.1016/j.jaci.2018.07.046 30240703 PMC6592617

[173] Hernandez-Pando R Jeyanathan M Mengistu G Aguilar D Orozco H Harboe M Persistence of DNA from Mycobacterium tuberculosis in superficially normal lung tissue during latent infection. Lancet. (2000) 356(9248):2133. 10.1016/S0140-6736(00)03493-0 11191539

[174] Giamarellos-Bourboulis EJ Tsilika M Moorlag S Antonakos N Kotsaki A Domínguez-Andrés J Activate: randomized clinical trial of BCG vaccination against infection in the elderly. Cell. (2020) 183(2):315–23.e9. 10.1016/j.cell.2020.08.051 32941801 PMC7462457

[175] Shirakawa T Enomoto T Shimazu S Hopkin JM. The inverse association between tuberculin responses and atopic disorder. Science. (1996) 275:77–9. 10.1126/science.275.5296.77 8974396

[176] Angelidou A Pittet LF Faustman D Curtis N Levy O. BCG Vaccine’s off-target effects on allergic, inflammatory, and autoimmune diseases: worth another shot? J Allergy Clin Immunol. (2022) 149(1):51–4. 10.1016/j.jaci.2021.09.034 34673049 PMC11688639

[177] Pittet LF Messina NL Gardiner K Freyne B Abruzzo V Francis KL Prevention of infant eczema by neonatal Bacillus Calmette-Guérin vaccination: the MIS BAIR randomized controlled trial. Allergy. (2021) 77(3):956–65. 10.1111/all.15022 34309859

[178] Maizels RM. Regulation of immunity and allergy by helminth parasites. Allergy. (2020) 75(3):524–34. 10.1111/all.13944 31187881

[179] Bilbo SD Wray GA Perkins SE Parker W. Reconstitution of the human biome as the most reasonable solution for epidemics of allergic and autoimmune diseases. Med Hypotheses. (2011) 77(4):494–504. 10.1016/j.mehy.2011.06.019 21741180

[180] Rook G Backhed F Levin BR McFall-Ngai MJ McLean AR. Evolution, human-microbe interactions, and life history plasticity. Lancet. (2017) 390(10093):521–30. 10.1016/S0140-6736(17)30566-4 28792414

[181] Arrais M Maricoto T Nwaru BI Cooper PJ Gama JMR Brito M Helminth infections and allergic diseases: systematic review and meta-analysis of the global literature. J Allergy Clin Immunol. (2022) 149(6):2139–52. 10.1016/j.jaci.2021.12.777 34968529

[182] Correale J Farez MF. The impact of parasite infections on the course of multiple sclerosis. J Neuroimmunol. (2011) 233(1–2):6–11. 10.1016/j.jneuroim.2011.01.002 21277637

[183] Fleming J Hernandez G Hartman L Maksimovic J Nace S Lawler B Safety and efficacy of helminth treatment in relapsing-remitting multiple sclerosis: results of the HINT 2 clinical trial. Multiple Sclerosis Journal. (2017) 25(1):81–91. 10.1177/1352458517736377 29064315 PMC5878983

[184] Charabati M Donkers SJ Kirkland MC Osborne LC. A critical analysis of helminth immunotherapy in multiple sclerosis. Mult Scler J. (2020) 26(12):1448–58. 10.1177/1352458519899040 31971074

[185] Ryan SM Eichenberger RM Ruscher R Giacomin PR Loukas A. Harnessing helminth-driven immunoregulation in the search for novel therapeutic modalities. PLoS Pathog. (2020) 16(5):e1008508. 10.1371/journal.ppat.1008508 32407385 PMC7224462

[186] Roslund MI Gronroos M Rantalainen AL Jumpponen A Romantschuk M Parajuli A Half-lives of PAHs and temporal microbiota changes in commonly used urban landscaping materials. PeerJ. (2018) 6:e4508. 10.7717/peerj.4508 29576975 PMC5863720

[187] Ugai T Sasamoto N Lee HY Ando M Song M Tamimi RM Is early-onset cancer an emerging global epidemic? Current evidence and future implications. Nat Rev Clin Oncol. (2022) 19(10):656–73. 10.1038/s41571-022-00672-8 36068272 PMC9509459

[188] Jones RB Alderete TL Kim JS Millstein J Gilliland FD Goran MI. High intake of dietary fructose in overweight/obese teenagers associated with depletion of Eubacterium and Streptococcus in gut microbiome. Gut Microbes. (2019) 10(6):712–9. 10.1080/19490976.2019.1592420 30991877 PMC6866686

[189] Marmot M Allen J Bell R Goldblatt P. Building of the global movement for health equity: from Santiago to Rio and beyond. Lancet. (2012) 379(9811):181–8. 10.1016/S0140-6736(11)61506-7 22014678

[190] Rook GAW Raison CL Lowry CA. Microbial “old friends”, immunoregulation and socioeconomic status. Clin Exp Immunol. (2014) 177(1):1–12. 10.1111/cei.12269 24401109 PMC4089149

[191] Ishaq SL Rapp M Byerly R McClellan LS O’Boyle MR Nykanen A Framing the discussion of microorganisms as a facet of social equity in human health. PLoS Biol. (2019) 17(11):e3000536. 10.1371/journal.pbio.3000536 31770370 PMC6879114

[192] Rook GAW. Evolution, the immune system, and the health consequences of socioeconomic inequality. mSystems. (2022) 7(2):e0143821. 10.1128/msystems.01438-21 35285679 PMC9040728

[193] Milcent C Zbiri S. Prenatal care and socioeconomic status: effect on cesarean delivery. Health Econ Rev. (2018) 8(1):7. 10.1186/s13561-018-0190-x 29525909 PMC5845483

[194] Uphoff E Cabieses B Pinart M Valdés M Antó JM Wright J. A systematic review of socioeconomic position in relation to asthma and allergic diseases. Eur Respir J. (2015) 46(2):364. 10.1183/09031936.00114514 25537562

[195] Noval Rivas M Chatila TA. Regulatory T cells in allergic diseases. J Allergy Clin Immunol. (2016) 138(3):639–52. 10.1016/j.jaci.2016.06.003 27596705 PMC5023156

[196] Yazdanbakhsh M van den Biggelaar A Maizels RM. Th2 responses without atopy: immunoregulation in chronic helminth infections and reduced allergic disease. Trends Immunol. (2001) 22:372–7. 10.1016/S1471-4906(01)01958-5 11429321

[197] ISAAC Steering Committee. Worldwide variation in prevalence of symptoms of asthma, allergic rhinoconjunctivitis, and atopic eczema: ISAAC. The international study of asthma and allergies in childhood (ISAAC) steering committee. Lancet. (1998) 351(9111):1225–32. 10.1016/S0140-6736(97)07302-9 9643741

[198] Whibley N Tucci A Powrie F. Regulatory T cell adaptation in the intestine and skin. Nat Immunol. (2019) 20(4):386–96. 10.1038/s41590-019-0351-z 30890797

[199] Osimo EF Pillinger T Rodriguez IM Khandaker GM Pariante CM Howes OD. Inflammatory markers in depression: a meta-analysis of mean differences and variability in 5,166 patients and 5,083 controls. Brain Behav Immun. (2020) 87:901–9. 10.1016/j.bbi.2020.02.010 32113908 PMC7327519

[200] Stearns SC. Evolutionary medicine: its scope, interest and potential. Proc Biol Sci. (2012) 279(1746):4305–21. 10.1098/rspb.2012.1326 22933370 PMC3479795

[201] Gluckman PD Hanson MA. Living with the past: evolution, development, and patterns of disease. Science. (2004) 305(5691):1733–6. 10.1126/science.1095292 15375258

